# Comparative Analysis of Pretreatment Methods for Processing Bulk Flax and Hemp Oilseeds Under Uniaxial Compression

**DOI:** 10.3390/foods14040629

**Published:** 2025-02-13

**Authors:** Abraham Kabutey, Sonia Habtamu Kibret, Asmerom Woldemichael Kiros, Meseret Abraham Afework, Michael Onwuka, Akshay Raj

**Affiliations:** Department of Mechanical Engineering, Faculty of Engineering, Czech University of Life Sciences Prague, 165 00 Prague, Czech Republic; xkibs001@studenti.czu.cz (S.H.K.); xkira011@studenti.czu.cz (A.W.K.); xafem001@studenti.czu.cz (M.A.A.); michaelonwuka26@gmail.com (M.O.); xraja009@studenti.czu.cz (A.R.)

**Keywords:** oilseeds, heating methods, drying temperatures, linear compression, regression models

## Abstract

The purpose of this study was to examine the effect of oven and vacuum pretreatment techniques at drying temperatures between 40 °C and 90 °C and a constant heating time of 60 min on the oil yield, energy output, and compressive stress of bulk flax and hemp oilseeds samples. The results showed that heating temperatures linearly increased the amounts of oil yield but did not correlate linearly with energy requirement. The oven pretreatment slightly increased the oil yield and energy compared to the vacuum pretreatment. Higher compressive stress values were observed for hemp oilseeds than flax oilseeds which could be attributed to the inherent structure of the oilseeds. Hemp oilseeds showed more toughness to compress than flax oilseeds which tend to have a softer texture. The lack-of-fit *p*-values > 0.05 of the linear regression models dependent on the heating temperature under both drying conditions indicate adequacy for predicting the calculated parameters. Tukey’s significance test showed that the means of oil yield and energy of bulk flax and hemp oilseeds under the oven and vacuum pretreatments revealed no significant difference implying that both pretreatment methods can initiate the same heat treatment effect on oil extraction efficiency with the corresponding energy requirement.

## 1. Introduction

Oilseed crops are one of the most abundant sources of vegetable oils which are produced and processed in large quantities and utilized in many applications including food, feed, oleochemicals, and biodiesel production. They contribute a significant role in human health due to their nutritional components [1,2,3]. The most cultivated oilseed crops are soybean, rapeseed, sunflower, peanut, cotton, and oil palm [4]. Recently, flaxseed (*Linum usitatissimum* L.) and hemp seed (*Cannabis sativa* L.) have become increasingly popular around the world because of their growing demand and economic benefits [5,6,7]. Flaxseed is rich in various functional components such as oil, protein, polysaccharides, and lignans [8,9,10]. It is an important oilseed crop grown mainly in Canada, Argentina, the USA, China, and India [5,11]. Flaxseed accounts for about 35–45% of oil located in the kernel composed of cotyledon and endosperm [5]. Flaxseed oil has various health-promoting benefits in decreasing cardiovascular diseases, reducing cancer mainly in the prostrate and mammary gland, it acts as a laxative effect, anti-inflammatory activity, osteoporosis alleviation, and menopausal symptoms [8]. Hemp seed oil is also nutritious and rich in essential fatty acids, linoleic acid, linolenic acid, omega-6, and omega-3 polyunsaturated fatty acids [7]. Hemp seeds contain about 30–50% oil with some variation between varieties depending on the climatic conditions and regions of cultivation [7,12,13]. Currently, hemp crops are not only utilized in the textile processing industry but also applied in the production of new materials, food processing, and medical and health products [14]. Hemp seeds oil have medicinal value, such as antioxidation, regulation of blood lipids, improvement of the digestive system, blood pressure reduction, aging delay, and brain nourishment [15,16]. The global market for industrial hemp will grow from USD 4.6 billion in 2019 to USD 9.4 billion in 2025 with an annual growth rate of 12.8% [17]. Most hemp is currently grown for its oil and biomass [12].

Mechanical pressing is commonly used for oil extraction from oilseeds. Its efficiency has improved over the years because of technological advancements in screw press design [1,2,7,18]. However, its percentage oil yield is still significantly lower compared to solvent extraction which adversely affects the economic feasibility of the extraction process [1]. Industrial processing of plant oils employs high-temperature (hot pressing) and cold pressing methods. A lower oil extraction rate is observed under cold pressing compared to hot pressing [7,18]. In the literature, pretreatment/drying of oilseeds using various methods has a significant influence on the output of oil and the quality attributes [5,19,20,21]. The drying process involves the principle of simultaneous heat and mass transfer and is quite an energy-intensive method [22]. Hot-air drying is the most used technique for agricultural products due to its feasibility and simplicity in operation, equipment design, and environmental requirements [21,23,24,25]. The vacuum drying method allows the use of low temperatures in the absence of oxygen which can present heat-sensitive and easily oxidizable foods [26]. Other pretreatment methods including microwave radiation, pulsed electric field, enzymatic treatment, ultrasonication, ohmic heating, and cold plasma are emerging drying technologies applied in food processing [3,23,27,28]. In addition, the instant controlled pressure-drop DIC technology provides an alternative pretreatment of natural structure for increasing the yields and enhancing the operation kinetics while preserving the final oil and cake quality [29]. Hybrid drying systems such as the combined vacuum and microwave are novel to help reduce post-harvest losses, delay deterioration, extend shelf life, and ensure rapidity, affordability, simplicity, and minimize energy consumption [21,30].

Pretreatment of oilseeds is a key factor determining the oxidative stability of edible oils. Oxidative deterioration of oils is of great concern in the oil industry and marketing. In highly oxidized oils, secondary oxidation products such as ketones and aldehydes can accumulate leading to potential toxic and carcinogenic effects. Due to this, considerable efforts are being made to reduce lipid oxidation during the processing and storage of edible oils [30,31,32,33,34]. Considering the nutritional health benefits of oilseeds such as flax and hemp, it is essential to explore the efficiency of the various pretreatment and oil extraction techniques that can give maximum oil extraction yield with an optimal energy requirement. The linear compression process involving a pressing vessel diameter with a plunger under a given load and speed has been used to process different oilseeds by describing the mechanical properties, force–deformation curves, oil output, and energy demand [35,36]. In comparison with the non-linear compression using mechanical screw press, all these properties cannot be described. The linear process thus provides an alternative approach for improving the mechanical screw press which is characterized by low oil yield and high oil content in the press cake. Information on the linear process has not been sufficiently studied on several oilseeds such as bulk flax and hemp oilseeds under different pretreatment methods. Therefore, the objective of the study was to examine different heating temperatures using the oven and vacuum drying methods for flax and hemp oilseeds by determining the percentage oil yield and energy demand under the linear compression process. The mechanical properties (compressive force, deformation, hardness, compressive stress, and secant modulus of elasticity) of bulk flax and hemp seeds were also examined.

## 2. Materials and Methods

### 2.1. Samples

Five kg samples of bulk brown flax and green hemp oilseeds were procured from Stredi, Prague, Czech Republic. Before the experimentation, the samples were kept under laboratory conditions at a temperature of 20 ± 1.54 °C and a humidity of 45 ± 2%.

### 2.2. Determination of Samples’ Moisture Content

The moisture content of the samples was determined using the conventional oven method at a drying temperature of 105 °C and a time of 17 h [37]. The electronic balance (KERN & SOHN 440–35, Balingen, Germany) with an accuracy of 0.01 g was used for the samples’ initial and final measurements. The moisture content of the samples was calculated according to Equation (1) [38] as follows:

(1) $$M_{C}=\frac{M_{SB}-M_{SA}}{M_{SB}}$$

where $M_{C}$ is the moisture content in wet basis (%), $M_{SB}$ is the mass of the sample before drying, and $M_{SA}$ is the mass of the sample after oven drying.

### 2.3. Pretreatments of Samples Under Oven and Vacuum Conditions

The laboratory temperature of 20 °C served as the control of the samples before the pretreatment process. The samples were pretreated at temperatures between 40 °C and 90 °C at a constant drying time of 60 min using a DS_Memmert Universal oven UF 110 (MEMMERT GmbH + Co. KG, Buechenbach, Germany) (Supplementary Materials Figure S1) and vacuum dryer (Goldbrunn 1450, Zielona Gora, Poland) (Supplementary Materials Figure S2). For the oven drying process, the drying time and the temperature were set using the control cockpit. The drying process time started when the preset temperature was reached. The air circulation during the drying process was controlled by setting the fan and restrictor air flap at 30%. The vacuum drying process was operated by first switching ON the vacuum pump at a 60 mbar. Afterwards, the vacuum dryer was switched ON and the preset temperature (SV) was set. When the measured temperature (PV) had reached the preset temperature, the sample was loaded into the dryer chamber. The vacuum gauge pressure was then set at 2.0 × 100 mbar before the drying process. The heating time was controlled by a digital stopwatch.

### 2.4. Extraction of Oil Under the Linear Compression Process

**Figure 1:**
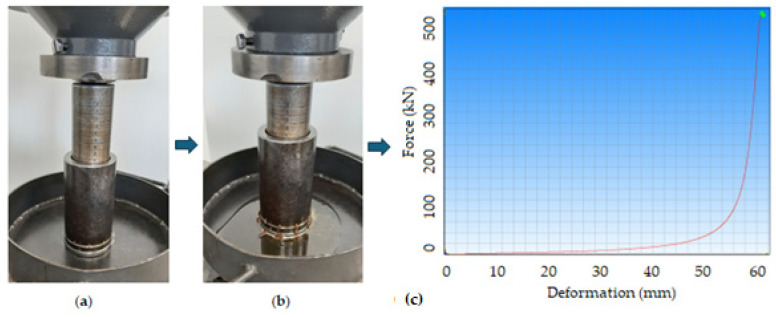
Linear compression process: (**a**) initial pressing height of sample, 80 mm before the compression test, (**b**) sample after the compression test showing the output oil, and (**c**) generated force-deformation curve as displayed in Figure 2 and Figure 3 for all the tests conducted. The green dot means the cessation of the compression process.

The extraction of the oil from the bulk oilseed samples was performed by utilizing the universal compression testing machine (TEMPOS spol. s.r.o., Opava, Czech Republic (Machine Service); ZDM 50, VEB Werkstoffprüfmaschinen, Leipzig, Germany) and the pressing vessel diameter of 60 mm with a plunger (Figure 1). The initial compression height of the samples was measured at 80 mm with the plunger corresponding to an initial mass of 171.07 g for flaxseeds and 127.3 g for hemp seeds. The volume of the samples was calculated to be 22.62 × 10^−4^ m^3^. The input load and the compression speed were 450 kN and 5 mm/min. The extraction process generated force–deformation curves data which were used to calculate the oil yield, energy, and mechanical properties (hardness, stress, and secant modulus of elasticity). The compression tests were repeated twice, and data were presented as mean ± standard deviation.

### 2.5. Percentage Oil Yield

The oil yield was calculated as the ratio of the mass of oil to the mass of the sample multiplied by 100 using Equation (2) [39,40] as follows:

(2) $$O_{YD}=\left[\left(\frac{M_{O}}{M_{S}}\right)\times 100\right]$$

where $O_{YD}$ is the oil yield (%), and $M_{O}$ is the mass of oil (g) calculated as the difference between the initial mass of the sample $M_{S}$ (g) and mass of pressed cake (g).

### 2.6. Energy Demand

The energy demand $E_{NG}$ is the area under the force–deformation curve which was calculated according to Equation (3) based on the trapezoidal rule [41,42,43,44] as follows:

(3) $$E_{NG}=\overset{n=i-1}{\underset{n=0}{\sum }}\left[\left(\frac{F_{n+1}+F_{n}}{2}\right)\times \left(X_{n+1}-X_{n}\right)\right]$$

where $E_{NG}$ is the energy demand (N·m = Joules, J), $F_{n+1}+F_{n}$ and $X_{n+1}-X_{n}$ are the force (N) the deformation (mm = 10^−3^ m), *n* is the number of data points and *i* is the number of sections in which the axis deformation was divided.

### 2.7. Statistical Analysis

The data were analyzed by employing the ANOVA and general linear regression techniques at a 0.05 significance level using Statistica software (version 13) [45] and Microsoft Excel (version 2411). The graphical descriptions were conducted using the Statistica software. Following the standard hypothesis test procedure, the null $H_{0}$ and alternative $H_{A}$ hypotheses of the study were specified as follows: $H_{0}$: there is no lack of fit in the regression model and $H_{A}$: there is a lack of fit in the regression model. If the *p*-value is smaller than the significance level alpha (α = 0.05), the null hypothesis is rejected in favor of the alternative. However, if the *p*-value is larger than the significance level alpha (α = 0.05), the null hypothesis is not rejected (that is the null hypothesis is accepted while the alternative is rejected, or the alternative hypothesis is not supported). The Tukey–Krammer procedure (Tukey’s test) was performed to test the significance or non-significance of the means of the calculated responses under oven and vacuum drying conditions about the effect of the heating temperature. In this case, for $H_{0}$, the means of the responses under oven and vacuum pretreatment conditions are equal, and for $H_{A}$, the means of the responses are unequal responses under oven and vacuum pretreatment conditions.

## 3. Results

### 3.1. Percentage Oil Yield and Energy Demand of Bulk Oilseeds

The calculated amounts of percentage oil yield and energy demand of bulk oilseeds (flax and hemp) under oven $O_{V}$ and vacuum $V_{C}$ pretreatment conditions for the heating temperatures are given in Table 1 and Table 2. The heating temperature of 20 °C served as the control whereas the heating temperatures between 40 °C and 90 °C were investigated. Based on the control result of bulk flax oilseeds under oven pretreatment, the percentage oil yield increased from 10.43 ± 0.32% to 20.84 ± 0.31% compared to the $V_{C}$ condition where the percentage oil yield increased from 10.43 ± 0.32% to 19.44 ± 0.50%. The energy demand increased from 847 ± 9.47 J to 1105.20 ± 142.44 J under $O_{V}$ and from 847 ± 9.47 J to 1100.24 ± 2.54 J under $V_{C}$. The percentage oil yield linearly increased under both drying conditions, but the energy demand showed both increasing and decreasing trends with the increase in heating temperature. The overall mean difference in oil yield increment under $O_{V}$ and $V_{C}$ was 0.88%. The corresponding energy was 19.77 J. For bulk hemp oilseeds under both drying conditions, the percentage oil yield increased from 20.23 ± 0.64% to 25.75 ± 0.07% in comparison with the $V_{C}$ where the percentage oil yield increased from 20.23 ± 0.64% to 25.05 ± 0.36%. On the other hand, the energy demand increased from 1410.05 ± 77.49 J to 1778.96 ± 92.77 J under $O_{V}$ and from 1410.05 ± 77.49 J to 1722.23 ± 121.69 J under $V_{C}$. The overall mean difference in oil yield increment under $O_{V}$ and $V_{C}$ was 0.42%. The corresponding energy was 55.74 J. It was observed that bulk hemp oilseeds produced a higher percentage of oil yield with a higher energy demand than bulk flax oilseeds under both drying conditions. This could be due to the inherent natural structure of the oilseed types.

### 3.2. Force–Deformation Curves of Bulk Oilseeds Under Pretreatment Conditions

**Figure 2:**
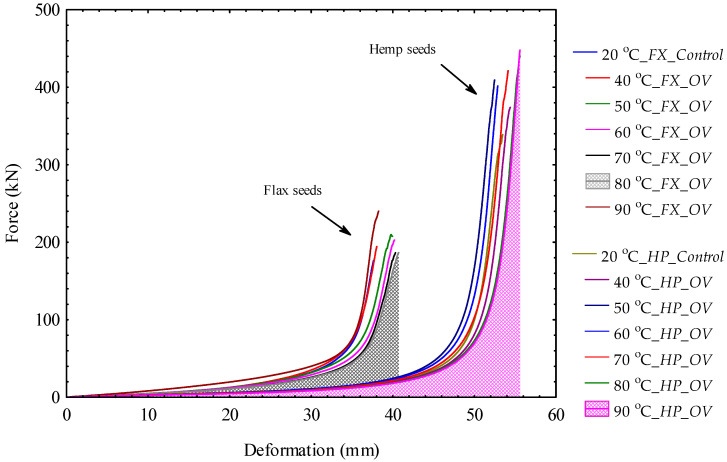
Comparison of force–deformation curves of bulk flax seeds, *FX*, and hemp seeds, *HP*, for the control and oven, $O_{V}$ heating temperatures (the area under the curve is the energy demand).

**Figure 3:**
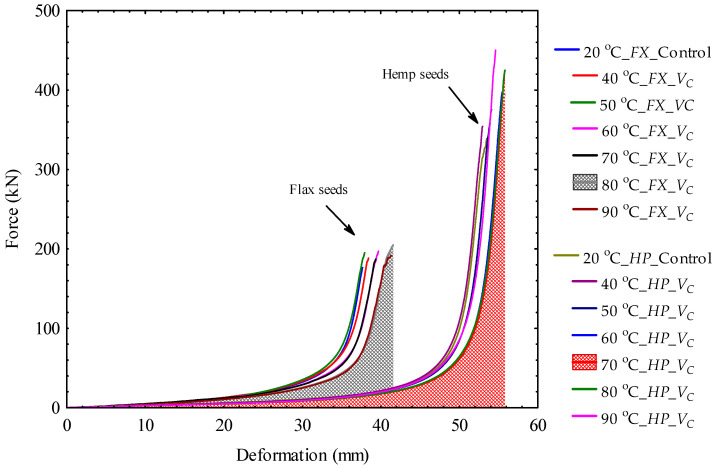
Comparison of force–deformation curves of bulk flax seeds, *FX*, and hemp seeds, *HP*, for the control and vacuum, $V_{C}$ heating temperatures (the area under the curve is the energy demand).

The force–deformation curves of bulk flax and hemp oilseeds under oven $O_{V}$ and vacuum $V_{C}$ heating temperatures are illustrated in Figure 2 and Figure 3. The area under the curve is the energy demand which was calculated according to Equation (3). For all the tests conducted, the compressive force values ranged from 174.97 ± 2.08 kN to 453.00 ± 3.97 kN with the corresponding deformation values from 37.70 ± 0.04 to 55.87 ± 0.40 mm. For bulk flax oilseeds under $O_{V}$, the maximum force of 211.60 ± 40.35 kN was observed at the heating temperature of 80 °C whereas the amount of 206.33 ± 1.78 kN was noticed at 90 °C under $V_{C}$. On the contrary, for bulk hemp oilseeds under both $O_{V}$ and $V_{C}$ conditions, the compressive force increased linearly with the increase in heating temperatures. The maximum force of 453.00 ± 3.97 kN was observed under $O_{V}$ whereas 443.54 ± 9.29 kN was produced under $V_{C}$. This result indicates that a higher force is required for compressing the oil from bulk hemp oilseeds than flax bulk oilseeds either at heating pretreatment or laboratory conditions. The compressive force is, however, related to the pressure, which is the ratio of the force to the cross-sectional area of the pressing chamber. The compressive force and the pressure values are presented in the Supplementary Materials Tables S1–S4 and further discussed in Section 4.

### 3.3. Mechanical Properties of Bulk Oilseeds Under Pretreatment Conditions

The mechanical properties of bulk flax and hemp oilseeds under oven $O_{V}$ and vacuum $V_{C}$ pretreatment conditions are presented in the Supplementary Materials (Tables S5–S8). The hardness values were calculated from the ratio of the force to the deformation [35,43]. The stress values were calculated from the ratio of the force to the cross-sectional area of the pressing chamber [35] whereas the secant modulus of elasticity values were calculated from the ratio of stress to the strain [35]. The deformation values were obtained directly from the compression experiments for the preset force and speed as well as the measured samples pressing height and the pressing chamber diameter.

For bulk flax seeds under $O_{V}$ condition (Table S5), the deformation values for the control temperature ranged from 37.70 ± 0.04 mm to 39.90 ± 2.30 mm. The calculated hardness values ranged from 4.64 ± 0.06 kN/mm to 5.34 ± 1.32 kN/mm. The stress values ranged from 61.88 ± 0.74 MPa to 74.84 ± 14.27 MPa and the secant modulus of elasticity values ranged from 98.49 ± 1.28 MPa to 113.36 ± 27.99 MPa. The values of the hardness, stress, and secant modulus of elasticity revealed that the bulk flax seeds became rigid at 40 °C and 90 °C. However, the rigidity declined from 50 °C to 80 °C, indicating that the heating temperatures do not have a direct effect on the toughness of flax seeds. Under $V_{C}$ condition (Table S6), the deformation values ranged 37.70 ± 0.04 mm to 41.40 ± 0.06 mm. The calculated hardness values ranged from 4.64 ± 0.06 kN/mm to 4.66 ± 0.05 kN/mm. The stress values ranged from 61.88 ± 0.74 MPa to 68.21 ± 0.76 MPa and the secant modulus of elasticity values ranged from 98.49 ± 1.28 MPa to 98.86 ± 0.96 MPa. The values of the hardness, stress, and secant modulus of elasticity revealed that the rigidity of bulk flax seeds increased from the control temperature of 20 °C until 60 °C and then declined at 90 °C.

For bulk hemp seeds under $O_{V}$ condition (Table S7), the deformation values for the control temperature ranged from 54.23 ± 0.99 mm to 55.87 ± 0.40 mm. The hardness values ranged from 6.22 ± 0.16 kN/mm to 8.11 ± 0.01 kN/mm. The stress values ranged from 119.26 ± 0.85 MPa to 160.21 ± 1.41 MPa and the secant modulus of elasticity values ranged from 131.98 ± 3.35 MPa to 172.07 ± 0.27 MPa. The hardness, stress, and secant modulus of elasticity linearly increased from the control temperature of 20 °C through to the heating temperatures between 40 °C and 70 °C. However, these parameters decreased at 80 °C and then increased at 90 °C. Under $V_{C}$ condition (Table S8), all the above-mentioned parameters increased linearly along with the heating temperatures with the control temperature except the deformation values which showed both increasing and decreasing trends. The deformation values ranged 54.23 ± 0.99 mm to 55.70 ± 0.15 mm. The hardness values ranged from 6.22 ± 0.16 kN/mm to 8.08 ± 0.23 kN/mm. The stress values ranged from 119.26 ± 0.85 MPa to 156.87 ± 3.29 MPa and the secant modulus of elasticity values ranged from 131.98 ± 3.35 MPa to 171.54 ± 4.94 MPa. The results showed that bulk hemp seeds demonstrated more robustness with heating treatment than flax seeds which tend not to be influenced by heating treatment.

### 3.4. Evaluation of ANOVA Analysis of Calculated Parameters

The ANOVA results of the percentage oil yield and energy of bulk flax and hemp oilseeds under oven $O_{V}$ and vacuum $O_{V}$ heating temperatures are presented in Table 3 and Table 4. The results show that under the pretreatment conditions the oil yield of bulk flax seeds (Table 3) significantly (*p*-value < 0.05) increased linearly with the heating temperature. The coefficient of determination (R^2^) values were between 0.963 and 0.974. The energy of flax seeds was also significantly influenced by the heating temperature under both pretreatment conditions with the R^2^ values between 0.301 and 0.578 indicating a correlation efficiency (R) between 54.88% and 76.05%. Similarly, the oil yield of bulk hemp seeds (Table 4) significantly (*p*-value < 0.05) increased linearly with heating temperature. The (R^2^) values were between 0.939 and 0.963. However, the increase in energy of hemp seeds under oven pretreatment was significant whereas under vacuum conditions it was non-significant (*p*-value > 0.05) since it showed both increasing and decreasing trends along with the heating temperature. The R^2^ values were between 0.758 and 0.876. This observation was further analyzed using the general linear model technique which otherwise indicated that the ANOVA results in Table 4 about the effect of heating temperature on the energy of bulk hemp seeds was rather significant.

The ANOVA results of the mechanical properties (hardness, compressive stress, and secant modulus of elasticity) of bulk flax and hemp oilseeds under oven $O_{V}$ and vacuum $O_{V}$ conditions are provided in the (Supplementary Materials Tables S9–S11). The compressive stress of bulk flax oilseeds under vacuum condition only showed significant (*p*-value < 0.05) with the heating temperature increment. However, all the mechanical properties of bulk hemp oilseeds under both oven $O_{V}$ and vacuum $O_{V}$ conditions showed significant (*p*-value < 0.05) with the heating temperature increment. The (R^2^) values ranged from 0.495 to 0.924.

### 3.5. Establishing a Linear Relationship Between the Predictor and the Responses

Based on the ANOVA results presented in Section 3.4, the determined regression models for predicting the responses (oil yield, energy, and secant modulus of elasticity) under oven and vacuum conditions as a function of heating temperature (predictor variable) are provided in Equations (4)–(13) as follows:

(4) $$O_{YD}\_O_{V}\_FX=8.016+0.145\times T_{PR}\;$$

(5) $$O_{YD}\_V_{C}\_FX=7.792+0.134\times T_{PR}\;$$

(6) $$E_{NG}\_O_{V}\_FX=856.828+2.289\times T_{PR}\;$$

(7) $$E_{NG}\_V_{C}\_FX=780.243+3.259\times T_{PR}\;$$

(8) $$O_{YD}\_O_{V}\_HP=18.278+0.086\times T_{PR}\;$$

(9) $$O_{YD}\_V_{C}\_HP=18.491+0.076\times T_{PR}\;$$

(10) $$E_{NG}\_O_{V}\_HP=1328.358+4.784\times T_{PR}\;$$

(11) $$E_{NG}\_V_{C}\_HP=1253.133+5.117\times T_{PR}\;$$

(12) $$E_{SM}\_O_{V}\_HP=126.2001+0.5428\times T_{PR}\;$$

(13) $$E_{SM}\_V_{C}\_HP=116.7689+0.6027\times T_{PR}\;$$

where $O_{YD}$: oil yield; $E_{NG}$: energy demand; $E_{SM}$: secant modulus of elasticity (MPa); $O_{V}$: oven drying; $V_{C}$: vacuum drying; flax (*FX*); hemp (*HP*); and $T_{PR}$: heating temperature.

**Figure 4:**
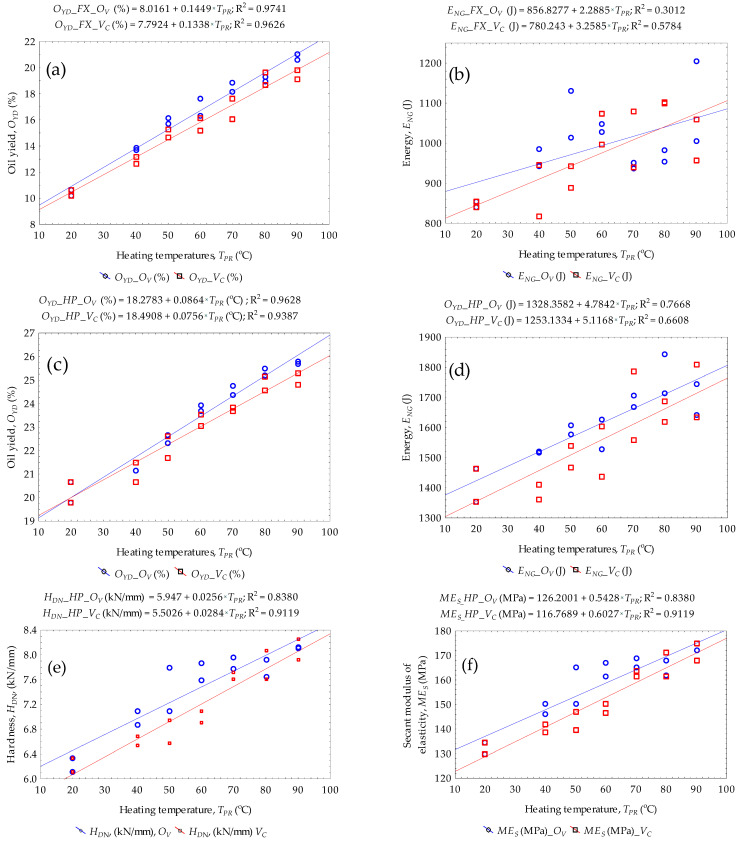
Scatterplots of (**a**) oil yield (**b**) energy for flax, (**c**) oil yield (**d**) energy for hemp oilseeds, and (**e**) hardness and (**f**) secant modulus of elasticity for hemp oilseeds.

The lack-of-fit *p*-values of the ANOVA results described in Table 3 and Table 4 above were greater than the probability level (α = 0.05) indicating that there is sufficient evidence to conclude that there is no lack of fit in the established regression models. The regression models thus correspond to the scatterplots depicted in Figure 4 explaining that the increase in the heating temperatures significantly (*p*-value < 0.05) increased the percentage oil yield and energy demand of bulk flax and hemp oilseeds under oven and vacuum pretreatment conditions. The other responses were the mechanical properties (deformation, compressive stress, hardness, and secant modulus of elasticity). There was a lack of fit (*p*-value < 0.05) in the regression models of the responses with the predictor variable for bulk flax oilseeds under both pretreatment conditions. However, for bulk hemp oilseeds, only the deformation parameter model indicated a lack of fit under oven pretreatment whereas the other responses showed no lack of fit (*p*-value > 0.05). Under vacuum pretreatment, all the responses proved no lack of fit. It is important to mention that since the calculated mechanical properties are interrelated then only the secant modulus of elasticity (rigidity of the pressing process) model is described in Equations (12) and (13) for bulk hemp oilseeds under oven and vacuum pretreatments.

### 3.6. Tukey–Krammer Significance Test of the Calculated Parameters

The calculated parameters (percentage oil yield, energy demand, compressive force/stress, hardness, and secant modulus of elasticity) under oven $O_{V}$ and vacuum $V_{C}$ were subjected to a homogeneous significance test by employing the Tukey–Krammer significance test (Tukey’s test) at α = 0.05. The outcome revealed that the means of oil yield and energy of bulk flax and hemp oilseeds under $O_{V}$ and $V_{C}$ pretreatments showed no significant difference implying that the means were equal (that is we failed to reject the null hypothesis since the *p*-value > 0.05). The analysis further implies that both pretreatment methods can induce the same heat treatment effect on oil extraction efficiency for the energy requirement. On the other hand, the means of the combined oil yield and energy of bulk flax and hemp oilseeds under $O_{V}$ and $V_{C}$ pretreatments showed that at least one of the means was not equal (that is we reject the null hypothesis in favor of the alternative hypothesis since the *p*-value < 0.05) indicating that the type of oilseed thus influences the percentage oilseed and the corresponding energy demand during the oil extraction process about pretreatment conditions. Similar outcomes were obtained for the mechanical properties. Detailed results of Tukey’s homogeneous group significance test are provided in the Supplementary Materials (Tables S12–S19). 

## 4. Discussion

In this study, the combined processing factors considered were the moisture content, compressive force (pressure), pressing speed, pressing vessel diameter, heating temperature, heating time, pretreatment methods, and oilseed type. The effect of these input factors on oil extraction efficiency, energy requirement, and mechanical properties (hardness, compressive stress, and secant modulus of elasticity) were examined. The obtained results and their statistical interpretation have been described in the preceding section. This section explains the results under the linear compression process in relation to the mechanical screw pressing by considering the input factors’ effect on the observed responses.

The moisture content of flaxseed and hemp seed was determined to be 6.18 ± 1.79 and 7.12 ± 0.91% w.b. representing the initial moisture content before the pretreatment process. However, after each pretreatment heating temperature, the moisture content was not determined before the oil extraction. Generally, the moisture content of oilseeds plays an important role in the oil extraction process. Lower moisture content of the seed increases friction whereas higher moisture content acts as a lubricant during the pressing operation [5,35,46,47,48]. Choking of the seeds or seedcake occurs with a lower seed moisture content. On the contrary, higher moisture content increases plasticity, thereby reducing the level of compression and contributing to poor oil recovery [35,48,49,50]. The quality of the oil is also affected by moisture content. A lower moisture content increases chlorophyll and phospholipids content in the oil. Conversely, sulfur, calcium, and magnesium contents in the oil increase at a higher moisture content [35,50,51,52]. The optimum moisture content varies among different oilseeds ranging between 6 and 15% wet basis [53,54,55]. The pressing speed was set constant at 5 mm/min. Higher pressing or screw speed results in higher speed of material throughput leading to higher residual oil content in the press cake since less time is available for the oil to drain from the solids [56,57]. At a higher speed, the viscosity thus remains lower resulting in less pressure build-up and more oil content in the press cake [56,57,58,59]. Other important input factors are the pressure and the heating temperature. In this study, the calculated pressure values increased linearly with heating temperatures for bulk hemp oilseeds under oven and vacuum pretreatment conditions compared to bulk flax oilseeds where the pressure values showed increasing and decreasing trends. In mechanical oil extraction processes, higher pressure will lead to higher temperature generation and higher oil recovery efficiency [58,59]. Theoretically, oil yield would be increased with the increase in heating temperature and pressure [57]. This statement thus confirms the oil yield amounts obtained in this study. However, it is reported that under a certain level, the increase in heating temperature and pressure will probably decrease the percentage of oil yield [39,60,61,62]. The authors further explained that the decrease in oil yield at higher heating temperatures could be due to the change in moisture content and structural changes in the oilseed material during the heating process. In addition, the increase in heating temperature to a certain level will reduce the moisture content of the seeds thus resulting in a reduction in water content. This reduction in moisture content will not be able to help in breaking/cracking the seed cell which thus results in lower percentage oil yield. In both linear and mechanical screw pressing operations, a smaller pressing vessel diameter or a smaller nozzle size and screw shaft diameter thus provide higher pressure towards the seeds for higher oil yield compared to bigger sizes or diameters which allow a larger space for the seeds to be filled, hence less pressure is provided towards the seeds resulting into lower oil yield [39]. In this study, under the linear pressing process, the pressing chamber or vessel diameter was 60 mm and it is suitable for oil extraction compared to a lower or higher diameter [35,36]. Regarding the oil recovered under the various heating temperatures and pretreatment conditions, it has been reported that heating or thermal treatments of oilseeds induce changes in microstructure as well as physical and chemical properties, physiochemical properties, and oxidative stability of the oil [31]. Cai et al. [31] mentioned that thermal treatment reduces the moisture content resulting in increased oil yield. In addition, color, flavor, and texture are enhanced, accessibility of bioactive compounds such as polyphenol and tocopherol are increased, and Maillard reaction products, for example, melanoidin, are formed, contributing to antioxidant activity which increases stability during storage and cooking [18,31,63,64,65]. These effects will be explored in future studies.

Finally, the oil yield and the energy demand with the processing factors are dependent on the inherent mechanical properties of the oilseeds. In linear oil pressing, the mechanical properties are the compressive force, deformation, hardness, compressive stress, and secant modulus of elasticity [36]. These properties among the oil extraction processes, physical, and chemical properties are essential for optimizing equipment design [54,66]. The ratio of the compressive force to that of the deformation is the hardness. The compressive force over the area of the pressing vessel or chamber is the compressive stress. The secant modulus of elasticity is the compressive stress over the strain (ratio of deformation to the initial pressing height of the bulk oilseeds). The higher values of the mechanical properties (Supplementary Materials Tables S5–S8) exhibited by the hemp bulk oilseeds showed that the shell of hemp seeds is harder than the flax bulk oilseeds which have a softer texture. This information is vital for deshelling efficiency which is subject to the compressive force/stress [66]. The results discussed herein are useful for achieving a higher efficiency of oil extraction from oil-bearing seeds.

## 5. Conclusions

The oil yield, energy requirement, and mechanical properties (hardness, stress, and secant modulus of elasticity) of bulk flax and hemp oilseeds were investigated under oven and vacuum pretreatment methods at heating temperatures between 40 °C and 90 °C. The oil extraction was performed using a linear compression process which generated force–deformation curves data for calculating the above-mentioned dependent parameters. The energy demand was calculated from the area under the curve based on the trapezoidal principle. The percentage oil yield linearly increased under both drying conditions, but the energy demand did not correlate linearly with the increase in heating temperature. Bulk hemp oilseeds produced a higher percentage oil yield, higher energy demand, and higher compressive stress than bulk flax oilseeds under both drying conditions which could be due to the inherent natural structure of the oilseed types. Tukey’s significance test showed that both pretreatment methods can initiate the same heat treatment effect on oil extraction efficiency, energy requirement, and the mechanical properties. The lack-of-fit *p*-values from the ANOVA analysis showed that the heating temperature significantly (*p* > 0.05) increased the percentage oil yield and energy requirement of bulk flax and hemp oilseeds. Linear regression models were described for the oil yield and energy amounts as a function of the heating temperature under the various drying methods. The models’ coefficients (heating temperature and the intercept) were significant (*p* < 0.05). The coefficients of determination (R^2^) of the established models ranged between 0.301 and 0.974. Regarding the mechanical properties of bulk flax oilseeds, only the stress parameter correlated positively with the heating temperature under vacuum condition with an R^2^ value of 0.495. The model’s coefficients (heating temperature and the intercept) were significant (*p* < 0.05). On the other hand, the mechanical properties of bulk hemp oilseeds under oven and vacuum conditions correlated positively with the heating temperature. The models’ coefficients (heating temperature and the intercept) were significant (*p* < 0.05) with R^2^ values between 0.929 and 0.977.

The findings of this present study are useful for setting the ideal input factors for processing different oilseeds with the mechanical screw press which is characterized by low oil yield and high oil content in the press cake. In future studies, the moisture content for each sample heating temperature will be determined before the oil extraction process. The oil quality analysis (oxidative stability of the oils or physicochemical properties) with the heating temperature under different pretreatment methods will be considered. In addition, the variation in the optimal processing factors will be optimized by employing optimization techniques such as the Box–Behnken design of the experiment coupled with the response surface regression technique.

## Figures and Tables

**Table 1:** Calculated $O_{YD}$ and $E_{NG}$ for flax oilseeds under $O_{V}$ and $V_{C}$ conditions.

$T_{PR}$ (°C)	Oven Pretreatment, $O_{V}$		Vacuum Pretreatment, $V_{C}$	
	$O_{YD}$ (%)	$E_{NG}$ (J)	$O_{YD}$ (%)	$E_{NG}$ (J)
20 *	10.43 ± 0.32	847 ± 9.47	10.43 ± 0.32	847 ± 9.47
40	13.76 ± 0.15	963.42 ± 30.41	12.91 ± 0.37	881.69 ± 91.86
50	15.95 ± 0.29	1072.19 ± 82.92	14.96 ± 0.38	916.27 ± 38.29
60	16.97 ± 0.95	1037.54 ± 15.23	15.64 ± 0.67	1035.31 ± 54.48
70	18.48 ± 0.53	943.16 ± 10.63	16.84 ± 1.10	1009.81 ± 98.11
80	19.11 ± 0.27	967.55 ± 20.25	19.19 ± 0.68	1100.24 ± 2.54
90	20.84 ± 0.31	1105.20 ± 142.44	19.44 ± 0.50	1007.36 ± 73.23

* Control at laboratory temperature; ±: standard deviation; $T_{PR}$: heating temperature; $O_{YD}$: oil yield; $E_{NG}$: energy demand.

**Table 2:** Calculated $O_{YD}$ and $E_{NG}$ for hemp oilseeds under $O_{V}$ and $V_{C}$ conditions.

$T_{PR}$ (°C)	Oven Pretreatment, $O_{V}$		Vacuum Pretreatment, $V_{C}$	
	$O_{YD}$ (%)	$E_{NG}$ (J)	$O_{YD}$ (%)	$E_{NG}$ (J)
20 *	20.23 ± 0.64	1410.05 ± 77.49	20.23 ± 0.64	1410.05 ± 77.49
40	21.16 ± 0.00	1518.83 ± 2.35	21.06 ± 0.58	1386.52 ± 35.33
50	22.49 ± 0.27	1592.46 ± 20.63	22.15 ± 0.64	1503.26 ± 49.30
60	23.82 ± 0.16	1578.53 ± 69.28	23.31 ± 0.34	1521.48 ± 116.92
70	24.57 ± 0.26	1686.06 ± 26.21	23.76 ± 0.08	1671.99 ± 162.40
80	25.35 ± 0.18	1778.96 ± 92.77	24.85 ± 0.42	1654.28 ± 48.39
90	25.75 ± 0.07	1695.12 ± 72.43	25.05 ± 0.36	1722.23 ± 121.69

* Control at laboratory temperature; ±: standard deviation; $T_{PR}$: temperature; $O_{YD}$: oil yield; $E_{NG}$: energy demand.

**Table 3:** ANOVA results of oil yield and energy of bulk flax oilseeds under $O_{V}$ and $V_{C}$ conditions.

Source	$O_{YD}$: Oil Yield (%) Under $O_{V}$: Oven Pretreatment				
	df	Sum of Squares	Mean Squares	F-Value	*p*-Value
$T_{PR}\;$(°C)	1	146.4650	146.4650	450.8847	0.0000 *
Residual Error	12	3.8981	0.3248		
Lack of Fit	5	2.3441	0.4688	2.1118	0.1786 **
Pure Error	7	1.553992	0.221999		
Total	13	150.3631			
**Source**	$O_{YD}$**: Oil Yield (%) Under** $V_{C}$ **: Vacuum Pretreatment**				
	**df**	**Sum of Squares**	**Mean Squares**	**F-Value**	***p*-Value**
$T_{PR}\;$(°C)	1	124.8500	124.8500	309.0554	0.0000 *
Residual Error	12	4.8477	0.4040		
Lack of Fit	5	2.0877	0.4175	1.0590	0.4549 **
Pure Error	7	2.7599	0.3943		
Total	13	129.6977			
**Source**	$E_{NG}$**: Energy (J) Under** $O_{V}$ **: Oven Pretreatment**				
	**df**	**Sum of Squares**	**Mean Squares**	**F-Value**	***p*-Value**
$T_{PR}\;$(°C)	1	36,510	36,510	5.1732	0.0421 *
Residual Error	12	84,691	7058		
Lack of Fit	5	55,756.14	11,151.23	2.6977	0.1141 **
Pure Error	7	28,935.28	4133.611		
Total	13	121,202			
**Source**	$E_{NG}$**: Energy (J) Under** $V_{C}$ **: Vacuum Pretreatment**				
	**df**	**Sum of Squares**	**Mean Squares**	**F-Value**	***p*-Value**
$T_{PR}\;$(°C)	1	74,021	74,021	16.4604	0.0016 *
Residual Error	12	53,963	4497		
Lack of Fit	5	26,007.08	5201.415	1.302422	0.3614 **
Pure Error	7	27,955.54	3993.649		
Total	13	12,7983			

$T_{PR}$: heating temperature, df: degrees of freedom; * *p*-Value < 0.05 denotes significant; ** *p*-Value > 0.05 denotes non-significant.

**Table 4:** ANOVA results of oil yield and energy of bulk hemp oilseeds under $O_{V}$ and $V_{C}$ conditions.

Source	$O_{YD}$: Oil Yield (%) Under $O_{V}$: Oven Pretreatment				
	df	Sum of Squares	Mean Squares	F-Value	*p*-Value
$T_{PR}\;$(°C)	1	52.0477	52.0477	310.6972	0.0000 *
Residual Error	12	2.0102	0.1675		
Lack of Fit	5	1.3929	0.2786	3.1591	0.0829 **
Pure Error	7	0.6173	0.0882		
Total	13	54.0580			
**Source**	$O_{YD}$**: Oil Yield (%) Under** $V_{C}$ **: Vacuum Pretreatment**				
	**df**	**Sum of Squares**	**Mean Squares**	**F-Value**	***p*-Value**
$T_{PR}\;$(°C)	1	39.8145	39.8145	183.610	0.0000 *
Residual Error	12	2.6021	0.2168		
Lack of Fit	5	1.0108	0.2022	0.8893	0.5353 **
Pure Error	7	1.5913	0.2273		
Total	13	42.4166			
**Source**	$E_{NG}$**: Energy (J) Under** $O_{V}$ **: Oven Pretreatment**				
	**df**	**Sum of Squares**	**Mean Squares**	**F-Value**	***p*-Value**
$T_{PR}\;$(°C)	1	159,563	159,563	39.4589	0.0000 *
Residual Error	12	48,525	4044		
Lack of Fit	5	22,751.01	4550.203	1.2358	0.3847 **
Pure Error	7	25,774.19	3682.027		
Total	13	208,088			
**Source**	$E_{NG}$**: Energy (J) Under** $V_{C}$ **: Vacuum Pretreatment**				
	**df**	**Sum of Squares**	**Mean Squares**	**F-Value**	***p*-Value**
$T_{PR}\;$(°C)	1	182,523	182,523	23.3756	0.0004 *
Residual Error	12	93,699	7808		
Lack of Fit	5	26,821.56	5364.313	0.561481	0.7281 **
Pure Error	7	66,877.10	9553.872		
Total	13	27,6221			

$T_{PR}$: heating temperature, df: degrees of freedom; * *p*-Value < 0.05 denotes significant; ** *p*-Value > 0.05 denotes non-significant.

## Data Availability

The original contributions presented in the study are included in the article; further inquiries can be directed to the corresponding author.

## Supplementary Materials

The following supporting information can be downloaded at www.mdpi.com/article/10.3390/foods14040629/s1, Figure S1: Standard Universal oven (UN 110) with a single display adaptive multifunctional digital PID-microprocessor controller with high-definition TFT-color display showing the varying heating temperatures (a)–(f) 40 °C and 90 °C during the drying process; Figure S2: Vacuum dryer Goldbrunn 1450 with a pump connection pipe showing the varying heating temperatures (a)–(f) 40 °C and 90 °C during the drying process; Table S1: Pressure amounts of bulk flax seeds under oven heating temperatures; Table S2: Pressure amounts of bulk flax seeds under vacuum heating temperatures; Table S3: Pressure amounts of bulk hemp seeds under oven heating temperatures; Table S4: Pressure amounts of bulk hemp seeds under vacuum heating temperatures; Table S5: Mechanical properties of bulk flax seeds under oven heating temperatures; Table S6: Mechanical properties of bulk flax seeds under vacuum heating temperatures; Table S7: Mechanical properties of bulk hemp seeds under oven heating temperatures; Table S8: Mechanical properties of bulk hemp seeds under vacuum heating temperatures; Table S9: Analysis of variance of compressive stress of bulk flax oilseeds under $V_{C}$; Table S10: Analysis of variance of mechanical properties of bulk hemp oilseeds under $O_{V}$ and Table S11: Analysis of variance of mechanical properties of bulk hemp oilseeds under $V_{C}$; Table S12: Tukey homogeneous group test of bulk flax seeds under oven $O_{V}$ temperatures; Table S13: Tukey homogeneous group test of bulk flax seeds under vacuum $V_{C}$ temperatures; Table S14: Tukey homogeneous group test of energy of bulk flax seeds under oven $O_{V}$ temperatures; Table S15: Tukey homogeneous group test of energy of bulk flaxseeds under $V_{C}$ temperatures; Table S16: Tukey homogeneous group test of oil yield of bulk hemp seeds under oven $O_{V}$ temperatures; Table S17: Tukey homogeneous group test of oil yield of bulk hemp seeds under $V_{C}$ temperatures; Table S18: Tukey homogeneous group test of energy of bulk hemp seeds under oven $O_{V}$ temperatures; Table S19: Tukey homogeneous group test of energy of bulk hemp seeds under $V_{C}$ temperatures.
