# Supplementary material for: Comparative Analysis of Pretreatment Methods for Processing Bulk Flax and Hemp Oilseeds Under Uniaxial Compression
Source: Foods. 2025 Feb 13;14(4):629. doi: 10.3390/foods14040629 (PMC11854633; doi:10.3390/foods14040629)
Supplement: Supplementary file 1 [file foods-14-00629-s001.zip › foods-3462546-supplementary.pdf]

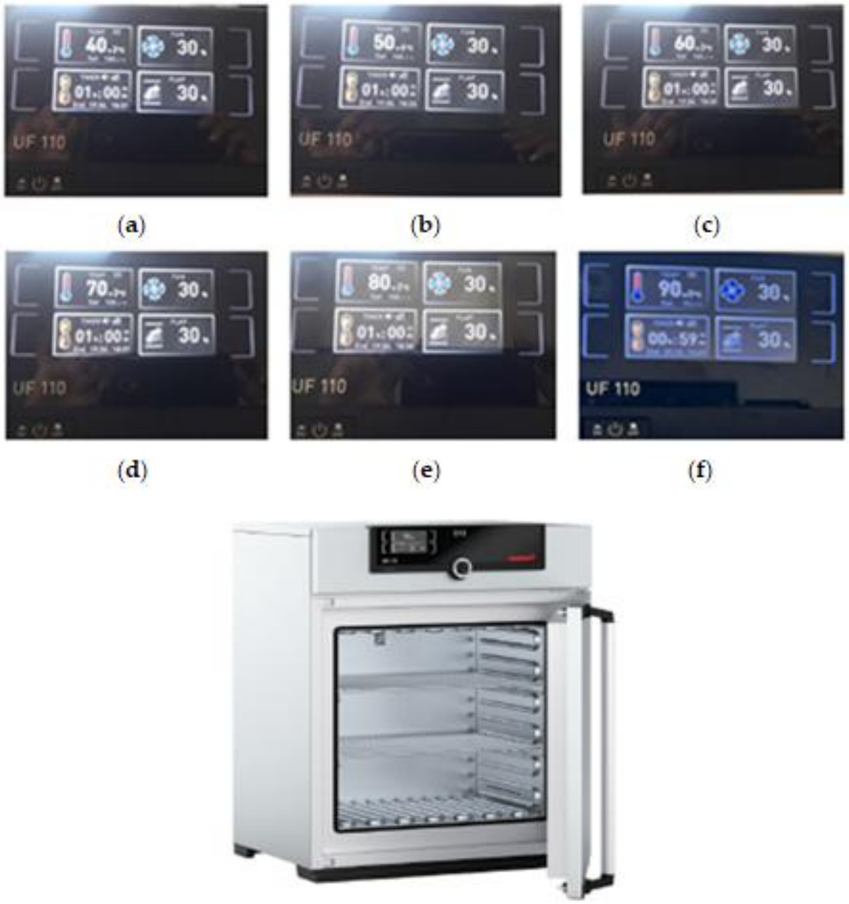

Figure S1. Standard Universal oven (UN 110) with a single display adaptive multifunctional digital PID-microprocessor controller with high-definition TFT-colour display showing the varying heating temperatures (a) – (f) 40 °C and 90 °C during the drying process.

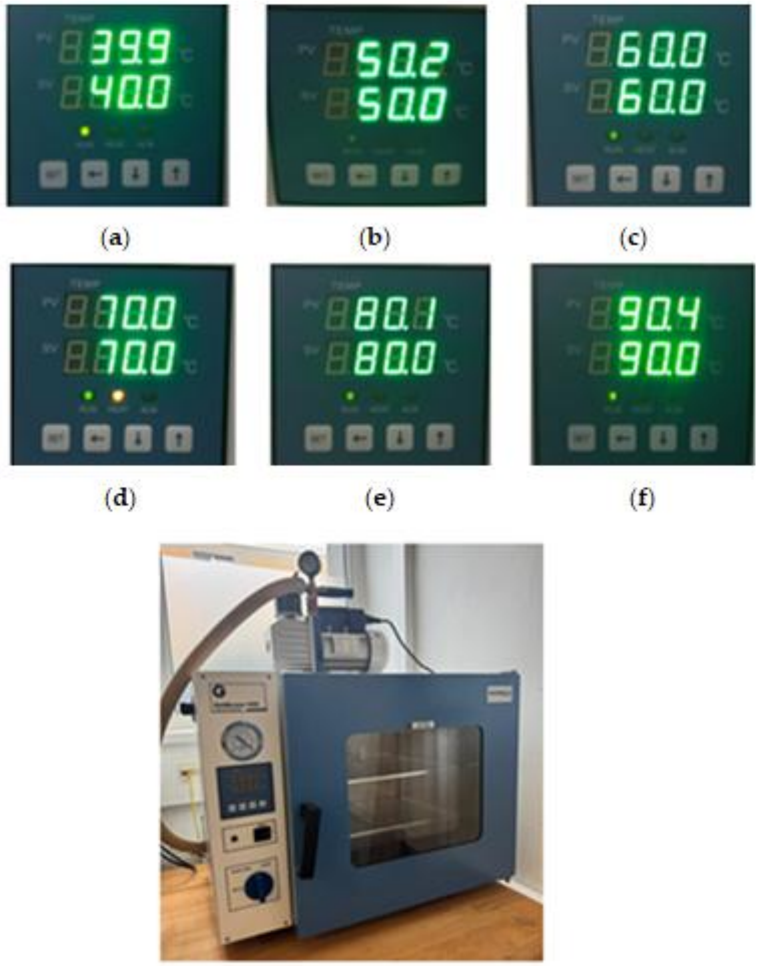

Figure S2. Vacuum dryer Goldbrunn 1450 with a pump connection pipe showing the varying heating temperatures (a) – (f) 40 °C and 90 °C during the drying process.

Table S1. Pressure amounts of bulk flax seeds under oven heating temperatures.

T_{PR} (°C)	F_{RC} (N)	F_{RC} (kN)	P_{TR} (MPa)
20*	174971.75 ± 2082.78	174.97 ± 0.04	61.88 ± 0.74
40	197276.75 ± 3860.45	197.28 ± 0.03	69.77 ± 1.37
50	203966.00 ± 5471.59	203.97 ± 0.47	72.14 ± 1.94
60	201025.25 ± 2519.07	201.03 ± 0.22	71.10 ± 0.89
70	186445.75 ± 98.64	186.45 ± 0.86	65.94 ± 0.03
80	185306.75 ± 1930.76	185.31 ± 0.42	65.54 ± 0.68
90	211600.50 ± 40352.46	211.60 ± 2.30	74.84 ± 14.27

* Control at laboratory temperature; ±: Standard deviation; T_{PR}: Heating temperature; F_{RC}: pressing force; P_{TR}: Pressure (ratio of F_{RC}/A); Cross-sectional area, A = 2827.433 mm^2

Table S2. Pressure amounts of bulk flax seeds under vacuum heating temperatures.

T_{PR} (°C)	F_{RC} (N)	F_{RC} (kN)	P_{TR} (MPa)
20*	174971.75 ± 2082.78	174.97 ± 0.04	61.88 ± 0.74
40	181708.75 ± 9365.28	181.71 ± 0.83	64.27 ± 3.31
50	190981.75 ± 5686.20	190.98 ± 0.28	67.55 ± 2.01
60	201379.50 ± 6243.75	201.38 ± 0.11	71.22 ± 2.21
70	198321.25 ± 16019.15	198.32 ± 1.48	70.14 ± 5.67
80	206332.75 ± 1778.73	206.33 ± 0.56	72.98 ± 0.63
90	192855.25 ± 2161.27	192.86 ± 0.06	68.21 ± 0.76

* Control at laboratory temperature; ±: Standard deviation; T_{PR}: Heating temperature; F_{RC}: pressing force; P_{TR}: Pressure (ratio of F_{RC}/A); Cross-sectional area, A = 2827.433 mm^2.

Table S3. Pressure amounts of bulk hemp seeds under oven heating temperatures.

T_{PR} (°C)	F_{RC} (N)	F_{RC} (kN)	P_{TR} (MPa)
20*	337210.00 ± 2401.33	337.21 ± 2.40	119.26 ± 0.85
40	376162.50 ± 2942.27	376.16 ± 2.94	133.04 ± 1.04
50	390720.50 ± 26308.61	390.72 ± 26.31	138.19 ± 9.30
60	407960.00 ± 8879.14	407.96 ± 8.88	144.29 ± 3.14
70	426902.75 ± 7760.14	426.90 ± 7.76	150.99 ± 2.74
80	427480.50 ± 17788.69	427.48 ± 17.79	151.19 ± 6.29
90	452996.75 ± 3972.88	453.00 ± 3.97	160.21 ± 1.41

* Control at laboratory temperature; ±: Standard deviation; T_{PR}: Heating temperature; F_{RC}: pressing force; P_{TR}: Pressure (ratio of F_{RC}/A); Cross-sectional area, A = 2827.433 mm^2.

Table S4. Pressure amounts of bulk hemp seeds under vacuum heating temperatures.

T_{PR} (°C)	F_{RC} (N)	F_{RC} (kN)	P_{TR} (MPa)
20*	337210.00 ± 2401.33	337.21 ± 2.40	119.26 ± 0.85
40	354008.00 ± 173.24	354.01 ± 0.17	125.20 ± 0.06
50	366716.25 ± 12234.72	366.72 ± 12.23	129.70 ± 4.33
60	387308.25 ± 11347.30	387.31 ± 11.35	136.98 ± 4.01
70	426751.00 ± 3925.86	426.75 ± 3.93	150.93 ± 1.39
80	432465.25 ± 10748.38	432.47 ± 10.75	152.95 ± 3.80
90	443541.75 ± 9292.44	443.54 ± 9.29	156.87 ± 3.29

* Control at laboratory temperature; ±: Standard deviation; T_{PR}: Heating temperature; F_{RC}: pressing force; P_{TR}: Pressure (ratio of F_{RC}/A); Cross-sectional area, A = 2827.433 mm^2.

Table S5. Mechanical properties of bulk flax seeds under oven heating temperatures.

T_{PR} (°C)	D_{FN} (mm)	H_{DN} (kN/mm)	σ_{SS} (MPa)	E_{SM} (MPa)
20*	37.70 ± 0.04	4.64 ± 0.06	61.88 ± 0.74	98.49 ± 1.28
40	38.06 ± 0.03	5.18 ± 0.11	69.77 ± 1.37	109.99 ± 2.23
50	40.32 ± 0.47	5.06 ± 0.19	72.14 ± 1.94	107.37 ± 4.12
60	40.09 ± 0.22	5.01 ± 0.04	71.10 ± 0.89	106.42 ± 0.75
70	39.80 ± 0.86	4.69 ± 0.10	65.94 ± 0.03	99.43 ± 2.10
80	40.43 ± 0.42	4.58 ± 0.00	65.54 ± 0.68	97.27 ± 0.01
90	39.90 ± 2.30	5.34 ± 1.32	74.84 ± 14.27	113.36 ± 27.99

* Control at laboratory temperature; ±: Standard deviation; T_{PR}: Heating temperature; D_{FN}: Deformation; H_{DN}: Hardness; σ_{SS}: Stress and E_{SM}: Secant modulus of elasticity.

Table S6. Mechanical properties of bulk flax seeds under vacuum heating temperatures.

T_{PR} (°C)	D_{FN} (mm)	H_{DN} (kN/mm)	σ_{SS} (MPa)	E_{SM} (MPa)
20*	37.70 ± 0.04	4.64 ± 0.06	61.88 ± 0.74	98.49 ± 1.28
40	37.83 ± 0.83	4.80 ± 0.14	64.27 ± 3.31	101.90 ± 3.01
50	38.13 ± 0.28	5.01 ± 0.19	67.55 ± 2.01	106.30 ± 3.95
60	39.68 ± 0.11	5.08 ± 0.17	71.22 ± 2.21	107.72 ± 3.63
70	40.44 ± 1.48	4.90 ± 0.22	70.14 ± 5.67	104.00 ± 4.61
80	41.20 ± 0.56	5.01 ± 0.11	72.98 ± 0.63	106.30 ± 2.36
90	41.40 ± 0.06	4.66 ± 0.05	68.21 ± 0.76	98.86 ± 0.96

* Control at laboratory temperature; ±: Standard deviation; T_{PR}: Heating temperature; D_{FN}: Deformation; H_{DN}: Hardness; σ_{SS}: Stress and E_{SM}: Secant modulus of elasticity.

Table S7. Mechanical properties of bulk hemp seeds under oven heating temperatures.

T_{PR} (°C)	D_{FN} (mm)	H_{DN} (kN/mm)	σ_{SS} (MPa)	E_{SM} (MPa)
20*	54.23 ± 0.99	6.22 ± 0.16	119.26 ± 0.85	131.98 ± 3.35
40	53.91 ± 0.71	6.98 ± 0.15	133.04 ± 1.04	148.10 ± 3.12
50	52.54 ± 0.01	7.44 ± 0.50	138.19 ± 9.30	157.81 ± 10.67
60	52.78 ± 0.13	7.73 ± 0.19	144.29 ± 3.14	164.04 ± 3.99
70	54.28 ± 0.14	7.86 ± 0.12	150.99 ± 2.74	166.89 ± 2.60
80	54.96 ± 0.89	7.78 ± 0.20	151.19 ± 6.29	165.02 ± 4.19
90	55.87 ± 0.40	8.11 ± 0.01	160.21 ± 1.41	172.07 ± 0.27

* Control at laboratory temperature; ±: Standard deviation; T_{PR}: Heating temperature; D_{FN}: Deformation; H_{DN}: Hardness; σ_{SS}: Stress and E_{SM}: Secant modulus of elasticity.

Table S8. Mechanical properties of bulk hemp seeds under vacuum heating temperatures.

T_{PR} (°C)	D_{FN} (mm)	H_{DN} (kN/mm)	σ_{SS} (MPa)	E_{SM} (MPa)
20*	54.23 ± 0.99	6.22 ± 0.16	119.26 ± 0.85	131.98 ± 3.35
40	53.56 ± 0.86	6.61 ± 0.11	125.20 ± 0.06	140.29 ± 2.31
50	54.32 ± 0.25	6.75 ± 0.26	129.70 ± 4.33	143.27 ± 5.45
60	55.34 ± 0.64	7.00 ± 0.12	136.98 ± 4.01	148.52 ± 2.62
70	55.70 ± 0.15	7.66 ± 0.09	150.93 ± 1.39	162.60 ± 1.93
80	55.21 ± 0.90	7.84 ± 0.32	152.95 ± 3.80	166.29 ± 6.84
90	54.88 ± 0.43	8.08 ± 0.23	156.87 ± 3.29	171.54 ± 4.94

* Control at laboratory temperature; ±: Standard deviation; T_{PR}: Heating temperature; D_{FN}: Deformation; H_{DN}: Hardness; σ_{SS}: Stress and E_{SM}: Secant modulus of elasticity.

Table S9. Analysis of variance of compressive stress of bulk flax oilseeds under V_C.

	σ_{SS}: Stress (MPa) under V_C: Vacuum pretreatment				
Source	df	Sum of squares	Mean Squares	F-Value	P-Value
T_{PR} (°C)	1	116.913	116.913	11.765	0.0049 *
Residual Error	12	119.247	9.937		
Lack of Fit	5	65.733	13.147	1.719	0.248 **
Pure Error	7	53.514	7.645		
Total	13	236.160			

T_{PR}: Heating temperature; df: degrees of freedom; * P-Value < 0.05 denotes significant and ** P-Value > 0.05 denotes non-significant.

Table S10. Analysis of variance of mechanical properties of bulk hemp oilseeds under O_V.

Source	H_{DN}: Hardness (**kN/mm**) under O_V: Oven pretreatment				
	df	Sum of squares	Mean Squares	F-Value	P-Value
T_{PR} (°C)	1	4.561	4.561	62.081	0.0000 *
Residual Error	12	0.882	0.073		
Lack of Fit	5	0.493	0.099	1.774	0.237 **
Pure Error	7	0.389	0.056		
Total	13	5.442			
Source	σ_{SS}: Stress (MPa) under O_V: Oven pretreatment				
	df	Sum of squares	Mean Squares	F-Value	P-Value
T_{PR} (°C)	1	2184.89	2184.89	139.842	0.0000 *
Residual Error	12	187.49	15.624		
Lack of Fit	5	40.154	8.031	0.382	0.847 **
Pure Error	7	147.334	21.048		
Total	13	2372.38			
Source	E_{SM}: Secant modulus of elasticity (MPa) under O_V: Oven pretreatment				
	df	Sum of squares	Mean Squares	F-Value	P-Value
T_{PR} (°C)	1	2053.67	2053.67	62.0810	0.0000 *
Residual Error	12	396.97	33.081		
Lack of Fit	5	221.885	44.377	1.774	0.237 **
Pure Error	7	175.081	25.012		
Total	13	2450.64			

T_{PR}: Heating temperature; df: degrees of freedom; * P-Value < 0.05 denotes significant and ** P-Value > 0.05 denotes non-significant.

Table S11. Analysis of variance of mechanical properties of bulk hemp oilseeds under V_C.

Source	H_{DN}: Hardness (**kN/mm**) under V_C: Vacuum pretreatment temperatures				
	df	Sum of squares	Mean Squares	F-Value	P-Value
T_{PR} (°C)	1	5.624	5.624	124.253	0.0000 *
Residual Error	12	0.543	0.045		
Lack of Fit	5	0.259	0.052	1.274	0.371
Pure Error	7	0.284	0.041		
Total	13	6.167			

Source	σ_{SS}: Stress (MPa) under V_C: Vacuum pretreatment temperatures				
	df	Sum of squares	Mean Squares	F-Value	P-Value
T_{PR} (°C)	1	2509.45	2509.45	145.728	0.0000 *
Residual Error	12	206.64	17.22		
Lack of Fit	5	143.905	28.781	3.211	0.080 **
Pure Error	7	62.736	8.962		
Total	13	2716.09			

Source	E_{SM}: Secant modulus of elasticity (MPa) under V_C: Vacuum pretreatment T_{PR}				
	df	Sum of squares	Mean Squares	F-Value	P-Value
T_{PR} (°C)	1	2532.59	2532.59	124.253	0.0000 *
Residual Error	12	244.59	20.38		
Lack of Fit	5	116.551	23.310	1.274	0.371 **
Pure Error	7	128.039	18.291		
Total	13	2777.18			

T_{PR}: Heating temperature; df: degrees of freedom; * P-Value < 0.05 denotes significant and ** P-Value > 0.05 denotes non-significant.

Table S12. Tukey homogeneous group test of bulk flax seeds under oven, O_V temperatures.

No.	T_{PR} (°C)	O_{YD} (%)	1	2	3	4	5	6
1	20	10.43					****	
2	40	13.76						****
3	50	15.95	****					
4	60	16.97	****	****				
5	70	18.48		****	****			
6	80	19.11			****	****		
7	90	20.84				****		

T_{PR}: Heating temperature; O_{YD}: Oil yield and **** not significantly different.

Table S13. Tukey homogeneous group test of bulk flax seeds under vacuum, V_C temperatures.

No.	T_{PR} (°C)	O_{YD} (%)	1	2	3	4	5
1	20	10.43		****			
2	40	12.91		****	****		
3	50	14.96	****		****		
4	60	15.64	****				
5	70	16.84	****			****	
6	80	19.19				****	****
7	90	19.44					****

T_{PR}: Heating temperature; O_{YD}: Oil yield and **** not significantly different.

Table S14. Tukey homogeneous group test of energy of bulk flax seeds under oven O_V temperatures.

No.	T_{PR} (°C)	E_{NG} (J)	1	2
1	20	847.00	****	
2	40	943.16	****	****
3	50	963.42	****	****
4	60	967.55	****	****
5	70	1037.54	****	****
6	80	1072.19	****	****
7	90	1105.20		****

T_{PR}: Heating temperature; E_{NG}: Energy demand and **** not significantly different.

Table S15. Tukey homogeneous group test of energy of bulk flaxseeds under V_C temperatures.

No.	T_{PR} (°C)	E_{NG} (J)	1	2
1	20	847.00	****	
2	40	881.69	****	****
3	50	916.27	****	****
4	60	1007.36	****	****
5	70	1009.81	****	****
6	80	1035.31	****	****
7	90	1100.24		****

T_{PR}: Heating temperature; E_{NG}: Energy demand and **** not significantly different.

Table S16. Tukey homogeneous group test of oil yield of bulk hemp seeds under oven O_V temperatures.

No.	T_{PR} (°C)	O_{YD} (%)	1	2	3	4
1	20	20.23		****		
2	40	21.16		****		
3	50	22.49				****
4	60	23.82			****	
5	70	24.57	****		****	
6	80	25.35	****			
7	90	25.75	****			

T_{PR}: Heating temperature; O_{YD}: Oil yield and **** not significantly different.

Table S17. Tukey homogeneous group test of oil yield of bulk hemp seeds under V_C temperatures.

No.	T_{PR} (°C)	O_{YD} (%)	1	2	3	4
1	20	20.23			****	
2	40	21.06			****	****
3	50	22.15		****		****
4	60	23.31	****	****		
5	70	23.76	****	****		
6	80	24.85	****			
7	90	25.05	****			

T_{PR}: Heating temperature; O_{YD}: Oil yield and **** not significantly different

Table S18. Tukey homogeneous group test of energy of bulk hemp seeds under oven O_V temperatures.

No.	T_{PR} (°C)	E_{NG} (J)	1	2	3
1	20	1410.05			****
2	40	1518.83	****		****
3	50	1578.53	****	****	****
4	60	1592.46	****	****	****
5	70	1686.06	****	****	
6	80	1695.12	****	****	
7	90	1778.96		****	

T_{PR}: Heating temperature; O_{YD}: Oil yield and **** not significantly different.

Table S19. Tukey homogeneous group test of energy of bulk hemp seeds under V_C temperatures.

No.	T_{PR} (°C)	E_{NG} (J)	1
1	20	1386.52	****
2	40	1410.05	****
3	50	1503.26	****
4	60	1521.48	****
5	70	1654.28	****
6	80	1671.99	****
7	90	1722.23	****

T_{PR}: Heating temperature; O_{YD}: Oil yield and **** not significantly different.
